# Forensic geophysics: ground penetrating radar (GPR) techniques and missing persons investigations

**DOI:** 10.1080/20961790.2019.1675353

**Published:** 2019-11-06

**Authors:** Pier Matteo Barone, Rosa Maria Di Maggio

**Affiliations:** a Archaeology and Classics Program, American University of Rome, Rome, Italy;; b Geoscienze Forensi Italia®-Forensic Geoscience Italy, Rome, Italy

**Keywords:** Forensic sciences, forensic geophysics, forensic archaeology, GPR, CSI

## Abstract

Ground penetrating radar (GPR) investigations have the potential to non-destructively detect buried or hidden targets and are therefore often used in forensic research. This study presents a particular application of GPR methods to search for a missing person in a specific subsurface environment: a natural cave. The search for missing people in Italy is a problematic and delicate task that needs improvement. Results of this study highlight not only the ability to detect both hollow and forensic targets, but also precisely locate and define their geometries. Moreover, GPR findings efficiently focus archaeological excavation and body recovery in an exact area and help to minimise time digging in erroneous places.

## Introduction

Ground penetrating radar (GPR) research is commonly used in crime scene investigations (CSI) worldwide owing to its rapid, precise and non-destructive technique (NDT) [1–7]. These advantageous characteristics make GPR an efficient and extensively used method to find missing people in different environments [8–13]. In Italy, only ∼65% of missing people are found after several searches [14]. The aim of this paper is to highlight the importance of NDT, such as geophysical methods, to help find missing people. We discuss how Italian law enforcement has used this system to find a missing person in the countryside of central Italy. Note that sensitive information is not released for privacy.

The missing person in question disappeared 6 months prior to the police request (June 2013). Owing to reliable intelligence, law enforcement was quite certain of the location where the person went missing. Specifically, a reinforced concrete road was relevant to this intelligence. Based on this information, the police had two hypotheses: (1) the person was killed and the reinforced concrete road was built to hide the body; or (2) after killing the person, the body was hidden in one of the many subsurface caves found in the area.

**Figure 1.**
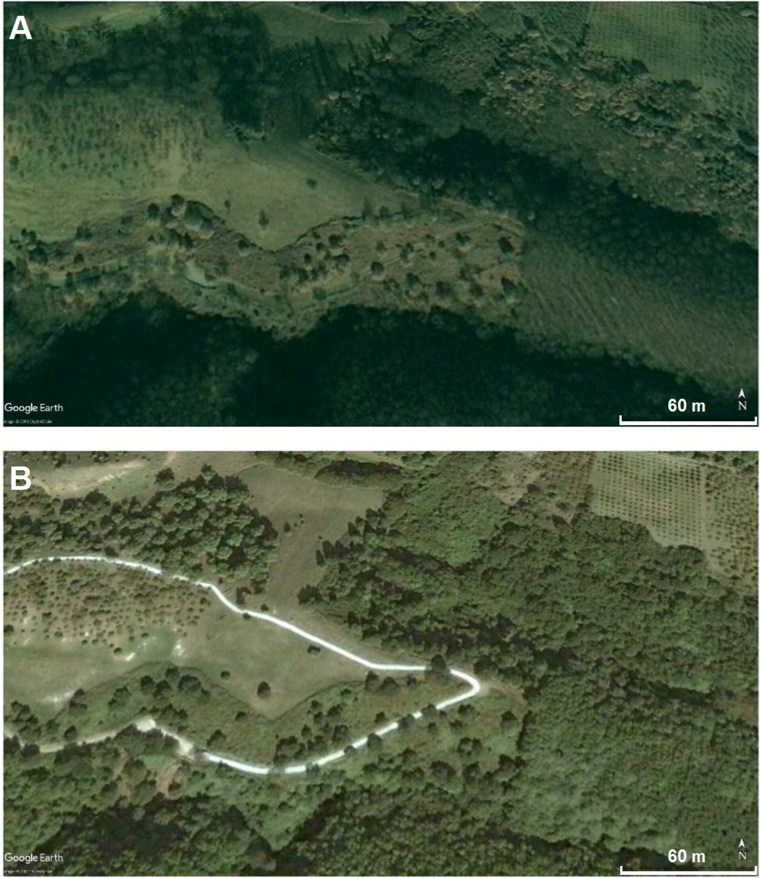
Comparison between two satellite images (between August 2004 (A) and November 2004 (B)) from Google Earth^®^ constrains the timing of the construction of the reinforced concrete road.

Before performing the GPR measurements, it was necessary to determine the construction date of the road, which was simplified with use of satellite images. In Figure 1, it is possible to see the year and approximate months in which the road was built: between August and November 2004. This comparison disproved hypothesis #1.

The next step was to collect GPR measurements in the above-mentioned area. Law enforcement’s second hypothesis was therefore followed owing to the particular geology of the region (volcanic tuff).

## Materials and methods

**Figure 2.**
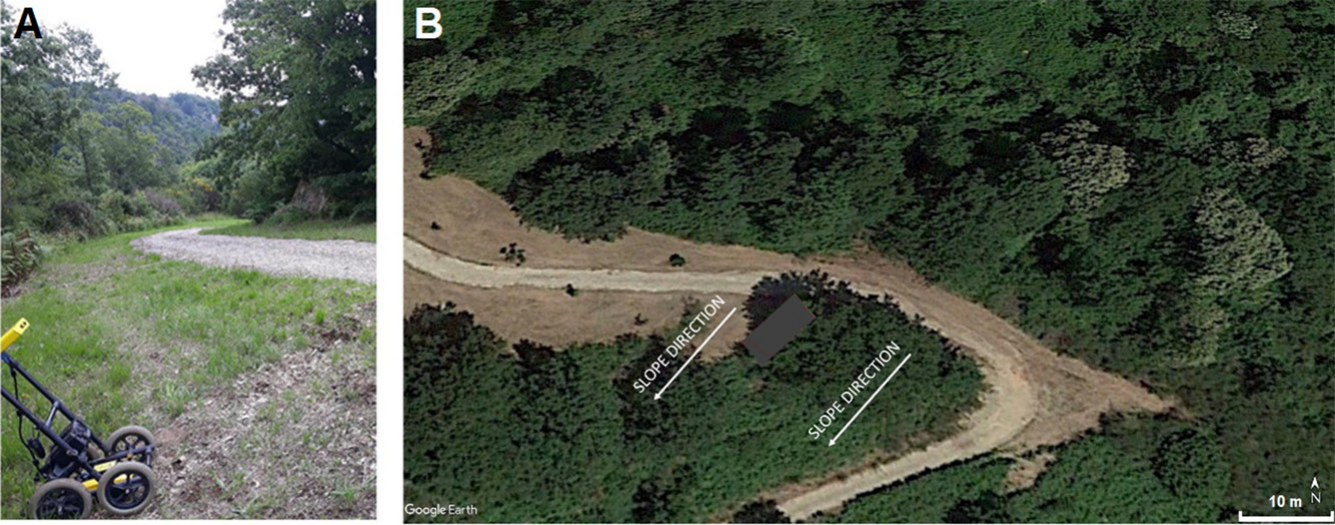
Area of the ground penetrating radar (GPR) measurements along the road. (A) A close-up is shown of the GPR system. (B) The satellite image with the investigated area (grey rectangle).

GPR was performed in the target area. The benefits of GPR to detect subsurface caves have been well documented [15–21]. Collected data used during this CSI were measured using the FINDAR system (Sensors & Software, Inc., Mississauga, Canada) with a bistatic GPR equipped with a 500-MHz antenna. This system was specifically designed for such forensic investigations and allows the operator to process the data in real time and on-site; a fundamental feature for law enforcement. Several parallel profiles were obtained with a line spacing of 0.5 m in the Y-direction (Y-grids). This acquisition produced several depth-slices using the average envelope amplitude algorithm and calibration hyperbola technique [22], generating an electromagnetic wave penetration velocity of 0.10 m/ns on the basis of the hyperbola calibration. Figure 2 shows how the GPR measurements were collected along the abovementioned road with particular regards to its edges.

## Results and discussion

**Figure 3.**
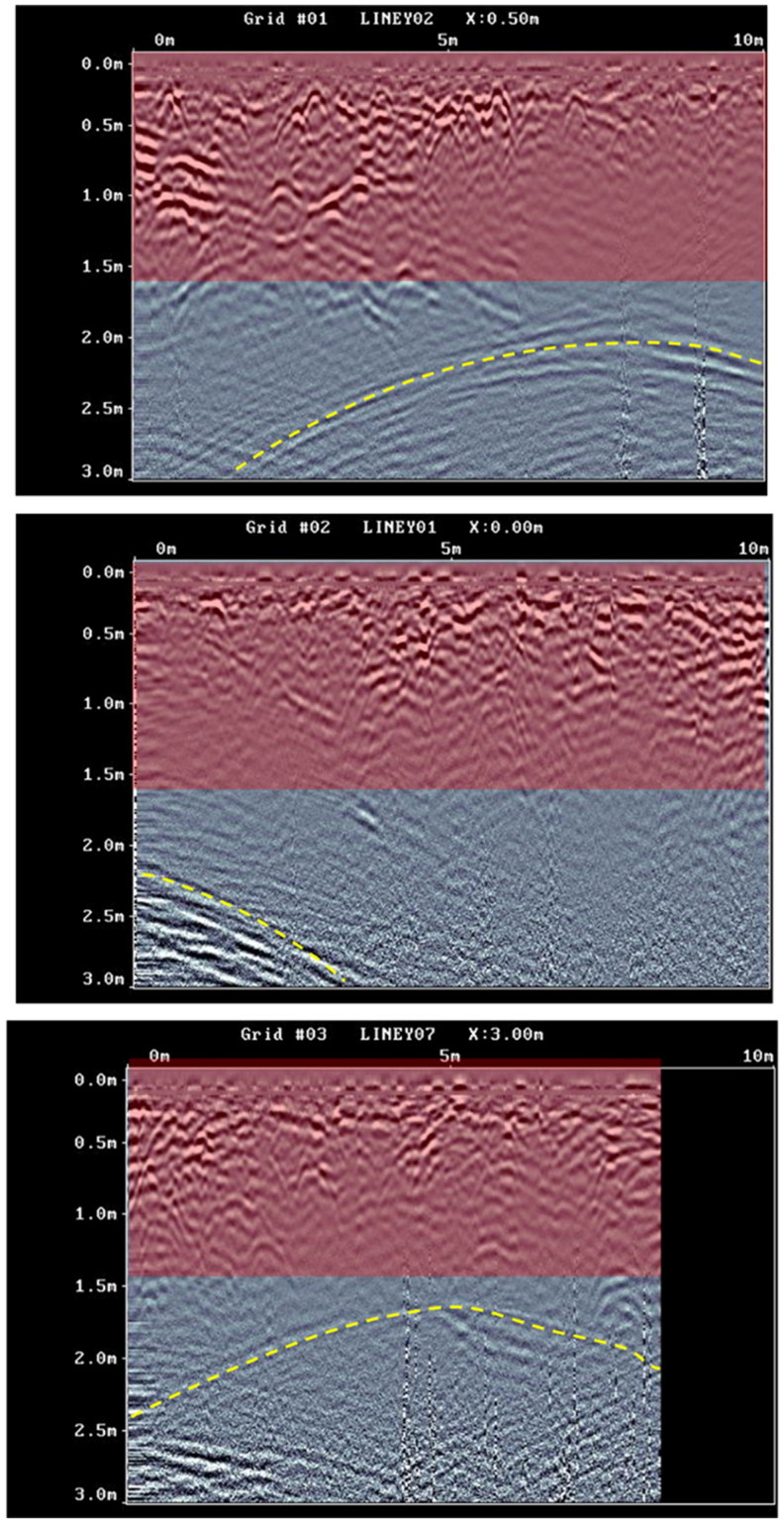
Three radargrams show roughly the area of a geological layer (red) at various depths around 2 m and interface between this layer and the curve roof of the subsurface cave (yellow).

Figure 3 shows three radargrams collected from different Y-grids. It is possible to identify an area interpreted as a geological layer created by local tuff blocks down to various depths around 2 m. The several hyperbolic events and discontinuities are as a result of the specific geomorphology of the rock [23]. Electromagnetic signatures from the road (i.e., anomalies) are not visible in these radargrams because they were collected beyond the road shoulder.

An interface with a typically curved geometry is evident starting at approximately 2 m depth and is visible in several radargrams at approximately this depth. The feature is easily interpreted as the top (i.e., roof) of a subsurface cave. However, a small number of radargrams from the same Y-grid highlight another strong anomaly unrelated to the presence of a cave.

**Figure 4.**
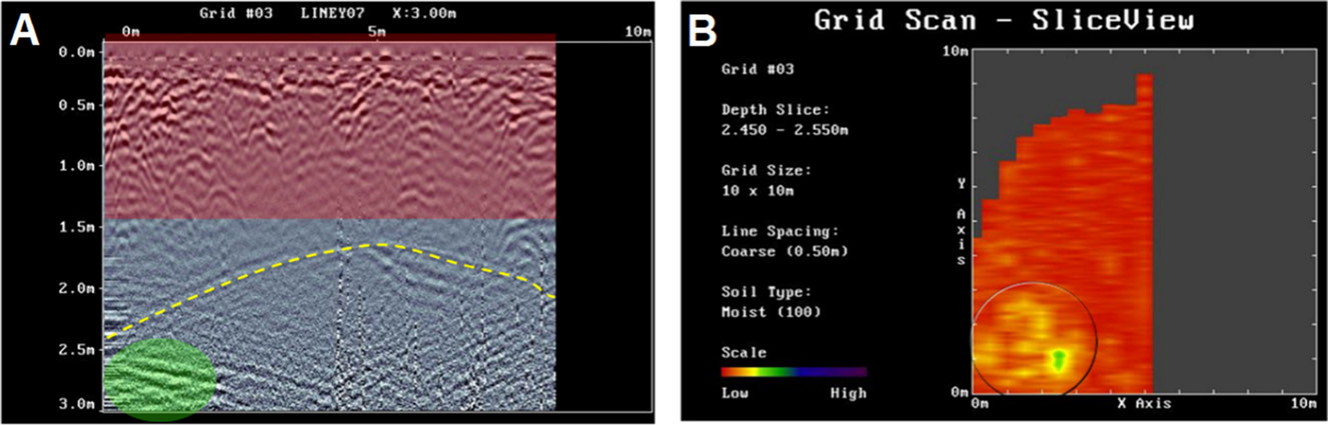
(A) The anomaly due to a possible body (green) is clearly below the roof. The depth is owing to a velocity of 0.10 m/ns of the above rough geological layer and is valid to a depth of 2 m. (B) The corresponding depth-slice in which the geometry of the cave (circled) and, in green/yellow, the same anomaly detected by the radargram are clear. The cave entrance was not covered by the ground penetrating radar (GPR) investigation.

Such an anomaly is evident in Figure 4(A) on the left side below the cave roof. Figure 4(B) further illustrates the crosscheck analysis of this anomaly through the depth-slice. The anomaly detected by the radargram above is clearly visible in yellow/green. The most relevant feature was interpreted as a possible anomaly caused by the corpse of the missing person owing to its focalised position in a specific place. In this depth-slice, it is also possible to observe the nearly circular geometry of the cave. This is because the cave is filled with air from the roof to the target. Air is clearly detectable by radio waves because it creates a strong reflector with a distinct dielectric constant.

**Figure 5.**
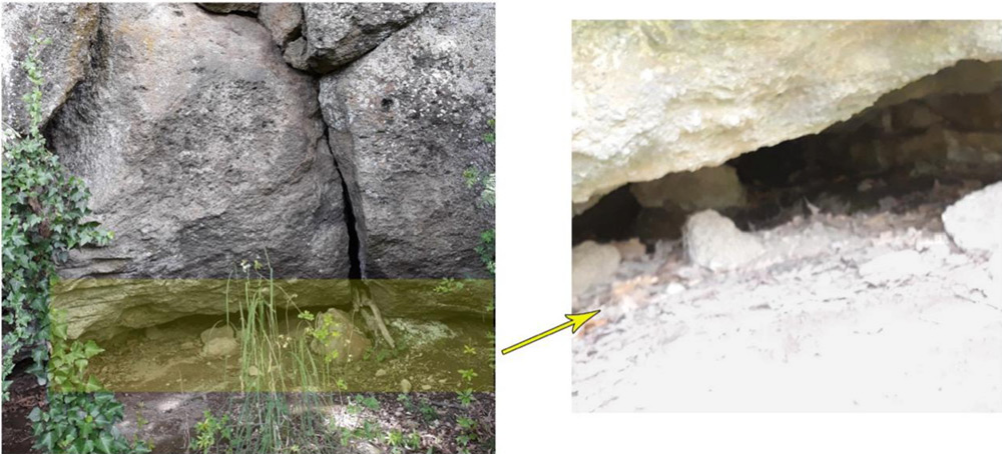
Images show how difficult it was to detect the entrance to the cavity. The magnified image on the right illustrates the inner part of the cavity beyond the obstructed access.

These very promising results and prescribed protocol [24, 25] prompted suggestion of an archaeological dig and recovery, however, the cave entrance needed to be located because the robust volcanic tuff rock negated the possibility of digging from the surface into the cave. We then began to search for access to the cave. The aim of the investigation at that point was to find lateral access to the cave covered by very dense vegetation. Once the cave entrance was located (Figure 5), it became clear that the offender had not only occluded the entrance with impenetrable shrubbery, but had also partially blocked it with stones and soil. Upon removal of these artificial obstacles, the inner cavern was opened and the body was found lying supine at the bottom of the cave in the exact position detected by the GPR, partially filled with debris.

## Conclusion

Because of the challenge of finding missing people, particularly in Italy [14], use of non-destructive methods, such as GPR, can tremendously assist forensic investigations and research. In many homicide cases, bodies are buried in the ground. In this case, we illustrate a specific CSI that detected a missing person as well as identified subsurface variations, such as caves, with indication of a buried body, facilitated by the particular geomorphology of the terrain. This method began with high-quality intelligence and analysis of large-scale satellite data to locate potential burial areas, such as detecting changes in the landscape. It was then possible to proceed with a GPR investigation. This non-destructive and rapid approach to a crime scene not only provided information of subsurface geological features, but also located the missing person’s body by analysing two main outputs: radargrams and depth-slices. The final stage involved carrying out a focused site search and digging according to archaeological methods.
